# The prognostic power of 18F-FDG PET/CT extends to estimating systemic treatment response duration in metastatic castration-resistant prostate cancer (mCRPC) patients

**DOI:** 10.1038/s41391-021-00391-8

**Published:** 2021-05-19

**Authors:** Matteo Bauckneht, Francesco Bertagna, Maria Isabella Donegani, Rexhep Durmo, Alberto Miceli, Vincenzo De Biasi, Riccardo Laudicella, Giuseppe Fornarini, Alfredo Berruti, Sergio Baldari, Annibale Versari, Raffaele Giubbini, Gianmario Sambuceti, Silvia Morbelli, Domenico Albano

**Affiliations:** 1 Nuclear Medicine, IRCCS Ospedale Policlinico San Martino, Genova, Italy; 2 Department of Health Sciences (DISSAL), University of Genova, Genova, Italy; 3 Nuclear Medicine, Spedali Civili of Brescia and University of Brescia, Brescia, Italy; 4 Nuclear Medicine, AUSL-IRCCS of Reggio Emilia, Reggio Emilia, Italy; 5 PhD Program in Clinical and Experimental Medicine, University of Modena and Reggio Emilia, Reggio Emilia, Italy; 6 Nuclear Medicine, Department of Biomedical and Dental Sciences and of Morpho-functional Imaging, University of Messina, Messina, Italy; 7 Medical Oncology Unit 1, IRCCS Ospedale Policlinico San Martino, Genova, Italy; 8 Department of Medical and Surgical Specialties, Radiological Sciences and Public Health, Medical Oncology Unit, Università degli Studi di Brescia, ASST Spedali Civili, Brescia, Italy

**Keywords:** Prognostic markers, Predictive markers, Cancer therapy

## Abstract

**Background:**

We aimed to test whether the prognostic value of 18 F‐Fluorodeoxyglucose Positron Emission Tomography/Computed Tomography (FDG-PET/CT) in metastatic castration-resistant prostate cancer (mCRPC) extends to the estimation of systemic treatment response duration.

**Methods:**

mCRPC patients submitted to FDG-PET/CT in four Italian centers from 2005 to 2020 were retrospectively enrolled. Clinical and biochemical data at the time of imaging were collected, and SUV max of the hottest lesion, total metabolic tumor volume (MTV), and total lesion glycolysis (TLG) were calculated. The correlation between PET- and biochemical-derived parameters with Overall Survival (OS) was analysed. The prediction of treatment response duration was assessed in the subgroup submitted to FDG-PET/CT in the six months preceding Chemotherapy (namely Docetaxel or Cabazitaxel, 24 patients) or Androgen-Receptor Targeted Agents (ARTA, namely Abiraterone or Enzalutamide, 20 patients) administration.

**Results:**

We enrolled 114 mCRPC patients followed-up for a median interval lasting 15 months. While at univariate analysis, prostate-specific antigen (PSA), Alkaline Phosphatase (ALP), MTV, and TLG were associated with OS, at the multivariate Cox regression analysis, the sole MTV could independently predict OS (*p* < 0.0001). In the subgroup submitted to FDG-PET/CT before the systemic treatment initiation, PSA and TLG could also predict treatment response duration independently (*p* < 0.05). Of note, while PSA could not indicate the best treatment choice, lower TLG was associated with higher success rates for ARTA but had no impact on chemotherapy efficacy.

**Conclusions:**

FDG-PET/CT’s prognostic value extends to predicting treatment response duration in mCRPC, thus potentially guiding the systemic treatment selection.

## Introduction

In the last years, the therapeutic strategy for advanced prostate cancer (PC) radically changed due to improved knowledge of PC basic biology and progression mechanisms. Indeed, new molecules have been registered for the metastatic castration-resistant phase of the disease (mCRPC), and several emerging compounds are currently in the path of their validation.

Since combinations of these drugs have not been successful [1, 2], the sequential strategy remains the dominant approach in the clinical setting. However, given that the treatment-related environmental stress may promote the selection of aggressive and cross-resistant cancer cell populations, it is reasonable that each line of treatment will affect the duration of response of the subsequent lines [3]. Group analyses showed that treatment sequencing optimization may favorably impact clinical outcomes in mCRPC patients [4–7]. Nevertheless, choosing the right time for the proper therapy is complicated at an individual patient level. No prospective sequencing or head-to-head comparison trials are available, as conducting these studies is challenging, making level 1 evidence lacking. Consequently, the patient’s or tumor’s characteristics or other clinical/biochemical parameters (such as the PSA value, the previous chemotherapy, the disease burden, or the presence of visceral metastases) derived from retrospective or phase 2 studies still represent the major determinants of treatment selection [8, 9]. In this scenario, there is an urgent need to identify biomarkers able to predict therapy effectiveness before its administration (thus potentially improving treatment selection on an individual basis).

Preclinical studies showed the occurrence of a metabolic glucose reprogramming in mCRPC patients developing resistance to androgen receptors (AR) targeted therapies [10, 11]. On the other hand, in the last years, several studies pointed out the potential prognostic value for 18 F‐Fluorodeoxyglucose Positron Emission Tomography/ Computed Tomography (FDG-PET/CT) [12–18] in prostate cancer patients, including the capability to predict time to hormonal failure in the castration-sensitive phase of the disease [17]. These findings suggest that tumor burden’s metabolic changes may potentially guide the treatment selection process in mCRPC.

On these bases, the present retrospective multicentric study aims to verify whether FDG-PET/CT’s predictive power extends to estimating systemic treatment response duration in mCRPC, thus potentially improving the treatment selection process.

## Patients and methods

### Study population

We performed a retrospective multicentric analysis of all consecutive mCRPC patients who underwent FDG-PET/CT from January 2005 to February 2020 in four Italian Institutes (IRCCS Ospedale Policlinico San Martino of Genoa, Spedali Civili of Brescia, AUSL-IRCCS of Reggio Emilia, and University of Messina). CRPC was defined as a serum testosterone level of <50 ng/dl following surgical or pharmaceutical castration. Recruited patients were submitted to FDG-PET/CT as indicated by the national guidelines [19]. Medical history, patient’s age, Gleason Score (GS), Prostate-specific antigen (PSA) at diagnosis, as well as PSA alkaline phosphatase (ALP), and lactate dehydrogenase (LDH) at the time of PET/CT, as well as the results of CT and bone scans performed near to FDG-PET/CT were collected.

According to the standard procedure of all centers, all patients signed a written informed consent form at the time of PET/CT, encompassing the use of anonymized data for research purposes. The local ethical committees approved the retrospective multicentric study.

### Imaging procedures and images analyses

FDG-PET/CT was performed according to the European Association of Nuclear Medicine (EANM) Guidelines [20]. Due to the retrospective design, PET/CT exams were acquired on different scanners, as detailed in Supplementary Table 1.

Maximum standardized uptake value (SUVmax) of the hottest metastatic lesion was obtained from FDG-PET/CT images. A volume of interest was then drawn using an SUV-based automated contouring program with an isocounter threshold based on 40% of the SUVmax [21]. The sum of all metastatic lesions identified the total Metabolic Tumor Volume (MTV). In contrast, the sum of the products between volume and the corresponding SUVmean of each lesion determined the Total Lesion Glycolysis (TLG).

### Statistical analysis

Descriptive analyses were conducted using percentages for binary variables and means/medians for continuous variables, reporting their dispersion values.

After the binarization of continuous variables, GS, serum variables (PSA, ALP, LDH), and FDG-PET/CT-derived parameters (SUVmax, MTV, TLG), were assessed for their correlation with Overall Survival (OS) as independent variables using a univariable and multivariable Cox regression model. The Youden index from the ROC curve for survival data was used to find the best cut-off value for all variables but GS and the number of bone scan lesions. GS at diagnosis was categorized into two classes for clinical interpretation, as previously described [22], while bone scan lesions were classified in < 6, 6–20, and > 20, as previously described [23]. All parameters with a *p* value < 0.10 at univariable analysis were selected for the multivariable analysis. OS was calculated from the time of FDG-PET/CT to death from any cause, censored at last follow-up for patients who were alive. Hazard-ratio (HR) for Cox regression models were reported together with a 95% confidence interval (CI) and *p* value. A *p* value of <0.05 was considered statistically significant. Survival curves were also generated using a Kaplan–Meier (KM) approach.

We thus focused on the subgroup of patients submitted to FDG-PET/CT in the six months preceding the start of systemic treatment (either Chemotherapy or Androgen Receptor-Targeted Agents, ARTA). The same Cox regression model was performed considering the Progression-Free Survival (PFS) as the final endpoint. PFS was calculated from FDG-PET/CT to the date of first disease progression, relapse, death, or the last follow-up date.

A Linear Regression analysis was performed using the Pearson correlation coefficient to explore further the association between the analyzed parameters and treatment response duration.

Analyses were conducted with IBM‐SPSS release 23 (IBM, Armonk, USA).

## Results

### Patients’ and treatment characteristics

114 mCRPC patients were retrospectively enrolled. Clinical, tumor, and treatment characteristics are summarized in Table 1.

**Table 1:** Clinical characteristics of the study population (*n* = 114).

Clinical characteristics	*n (%)*
Median age, years (range)	73.62 (51.6–88.7)
Gleason score at diagnosis	
≤7	56 (49.9%)
≥8	58 (50.1%)
Prostatectomy	
Yes	40 (35.1%)
No	72 (63.2%)
Missing data	2 (1.7%)
Lymphadenectomy	
Yes	33 (28.9%)
No	75 (65.8%)
Missing data	6 (5.3%)
Radical radiotherapy	
Yes	11 (9.6%)
No	101 (88.6%)
Missing data	2 (1.8%)
Metastatic disease at diagnosis	
Yes	62 (54.3%)
No	51 (44%)
Missing data	1 (1.7%)
Median PSA at diagnosis, ng/mL (range)	23 (1–6471)
Median ALP at diagnosis, U/L (range)	150.5 (33–613)
Median LDH at diagnosis, U/L (range)	234.5 (17–497)
Median interval between diagnosis and PET/CT, years (range)	5.6 (0.19–22.1)
Lines of treatment for CRPC at the time of FDG PET/CT	
1	22 (19.3%)
2	31 (27.2%)
≥2	58 (50.9%)
Missing data	3 (2.6%)
Prior chemotherapy	
Yes	64 (56.1%)
Docetaxel	46 (40.3%)
Cabazitaxel	18 (15.8%)
No	47 (41.2%)
Missing data	3 (2.6%)
Site of Metastases at the time of FDG PET/CT	
Exclusive lymph node metastases	3 (2.6%)
Bone and lymph node metastases	95 (83.4%)
Visceral metastases	16 (14%)
N° bone metastases at the time of FDG PET/CT	
<6	18 (15.7%)
6–20	40 (35.1%)
>20	32 (28.1%)
Missing data	24 (21.1%)
Median PSA at the time of FDG PET/CT, ng/mL (range)	64 (0.02–6471)
Median ALP at the time of FDG PET/CT, U/L (range)	100 (30–1266)
Median LDH at the time of FDG PET/CT, U/L (range)	271.2 (23–2349)

*PSA* prostate-specific antigen, *ALP* alkaline phosphatase, *LDH* lactate dehydrogenase, *CRPC* castration-resistant prostate cancer.

All patients had a histological diagnosis of PC with a median GS of 8 (range 5–10) with a GS ≥ 8 in 50.1% of patients; 54.3% of patients had metastatic disease at diagnosis. At the time of FDG-PET/CT, the median age was 81.6 years (range 52.7-88.7 years). CT revealed the occurrence of lymph node-only metastases in 2.6% of patients, while lymph node and bone metastases were present in 83.4% of patients. Visceral metastases were detected in 14% of patients. Most of the enrolled patients underwent FDG imaging after at least two systemic treatment lines for CRPC (50.8%), including chemotherapy (56.1%).

### Overall survival analysis

All patients included in the study were assessable for overall survival analysis and were followed-up for a median of 15 months (range: 0.8–42 months). The median OS (mOS) was 12.7 months, with 6 months and 12 months-OS of 52% (Standard Error: 0.05) and 36% (Standard Error: 0.06), respectively. Supplementary Fig. 1 shows the Kaplan–Meier survival function of the study cohort. Results from univariable and multivariable Cox regression analyses are reported in Table 2.

**Table 2:** Clinical characteristics and FDG-derived parameters in the prediction of OS in the whole cohort (*n* = 114).

	Univariate		Multivariate	
	HR (95% CI)	*p* value	HR (95% CI)	*p* value
*Patients’ characteristics*				
Gleason score				
≤7	1.00 (ref)	–		
≥8	1.07 (0.66–1.74)	0.77		
Metastatic disease at diagnosis				
No	1.00 (ref)			
Yes	1.31 (0.81–2.13)	0.26		
PSA at diagnosis				
≤22.5 ng/mL	1.00 (ref)	–		
>22.5 ng/mL	1.470 (0.805–2.685)	0.21		
ALP at diagnosis				
≤67.5 IU/L	1.00 (ref)	–		
>67.5 IU/L	2.448 (0.317–18.89)	0.39		
LDH at diagnosis				
≤223.5 IU/L	1.00 (ref)	–		
>223.5 IU/L	0.608 (0.190–1.945)	0.402		
Lines of treatment for CRPC at the time of PET/CT				
1	1.00 (ref)	–		
≥2	1.37 (0.79–2.39)	0.25		
Prior chemotherapy				
No	1.00 (ref)	–		
Yes	1.55 (0.76–3.15)	0.22		
Site of Metastases at the time of PET/CT				
Exclusive lymph node	1.00 (ref)	–		
Bone and lymph node	1.79 (0.24–13.02)	0.56		
Visceral	2.12 (0.26–16.73)	0.47		
No of bone metastases				
<6	1.00 (ref)	–		
6–20	1.95 (0.83–4.58)	0.12		
≥20	3.13 (1.39-7.03)	**0.006**		
PSA at PET/CT				
≤58.3 ng/mL	1.00 (ref)	–		
>58.3 ng/mL	3.996 (2.201–7.257)	**0.0001**	2.161 (1.014–4.606)	**0.046**
ALP at PET/CT				
≤45.5 IU/L	1.00 (ref)	–		
>45.5 IU/L	1.666 (0.662–4.196)	0.278		
LDH at PET/CT				
≤214.5 IU/L	1.00 (ref)	–		
>214.5 IU/L	0.830 (0.247–2.787)	0.763		
***FDG-PET parameters***				
SUV max (1-unit)				
≤6.9	1.00 (ref)	–		
>6.9	1.221 (0.747–1.997)	0.426		
MTV (1-unit)				
≤325.97 cm^3^	1.00 (ref)	–		
>325.97 cm^3^	2.288 (1.404–3.728)	**0.001**	2.647 (1.270–5.518)	**0.009**
TLG (1-unit)				
≤844.86	1.00 (ref)	–		
>844.86	2.208 (1.345–3.626)	**0.002**		

*PSA* prostate-specific antigen, *ALP* alkaline phosphatase, *LDH* lactate dehydrogenase, *CRPC* castration-resistant prostate cancer, *SUV* standardized uptake value, *MTV* metabolic tumor volume, *TLG* total lesion glycolysis.

High PSA and ALP serum levels as well as the presence of ≥20 bone metastases at the time of PET/CT were associated with lower OS. On the other hand, lower MTV and TLG correlated with an increased OS. Obtained cut-off values from the ROC curve analysis for OS for these variables are reported in Supplementary Table 2. Kaplan–Meier curves for these parameters after the binarization of continuous variables are reported in Supplementary Fig. 2. MTV remained also independently associated with OS at the multivariate analysis (*p* < 0.0001).

### Systemic treatment response duration

From the initial group of 114 mCRPC, we identified 44 patients submitted to FDG-PET/CT in the six months preceding systemic treatment initiation with either Chemotherapy (Docetaxel or Cabazitaxel, *n* = 24) or ARTA (Abiraterone or Enzalutamide, *n* = 20). The selection process details are reported in the Supplementary Fig. 3. No significant differences were observed between the obtained subgroups regarding clinical, tumor, and imaging characteristics (Supplementary Table 3).

These patients were clinically followed-up for a median interval of 18 months (range 0.8–42). At the survival analysis, the mOS of the entire group was 16.9 months, with 6 months and 12 months-OS of 68% (Standard Error: 0.08) and 46% (Standard Error: 0.1), respectively. The subgroup of patients undergoing ARTA showed an mOS of 19.1 months, while mOS was 14.4 months in patients undergoing Chemotherapy. However, this difference was not significant at the Log rank test (Supplementary Fig. 4A). The median PFS (mPFS) was 7.2 months, with 6 months and 12 months-PFS of 21% (Standard Error: 0.07) and 7% (Standard Error: 0.05), respectively. Again, mPFS was not significantly different between the ARTA and Chemotherapy subgroups, resulting 9.2 and 6.1 months, respectively (Supplementary Fig. 4B).

At the univariate analysis, PSA at the time of PET/CT, MTV, and TLG significantly predicted the systemic treatment response duration (PFS). Among them, TLG remained independently associated with PFS at the multivariate analysis (Table 3).

**Table 3:** Clinical characteristics and FDG-derived parameters in PFS prediction in the subgroup with pre-treatment PET (*n* = 44).

	Univariate		Multivariate	
	HR (95% CI)	*p* value	HR (95% CI)	*p* value
*Patients’ characteristics*				
Gleason score				
≤7	1.00 (ref)	–		
≥8	1.09 (0.54–2.16)	0.80		
Metastatic disease at diagnosis				
No	1.00 (ref)			
Yes	1.36 (0.40–4.53)	0.61		
PSA at diagnosis				
≤18.1 ng/mL	1.00 (ref)	–		
>18.1 ng/mL	0.82 (0.38–1.79)	0.629		
ALP at diagnosis				
≤150 IU/L	1.00 (ref)	–		
>150 IU/L	0.26 (0.05–1.36)	0.113		
LDH at diagnosis				
≤262 IU/L	1.00 (ref)	–		
>262 IU/L	0.45 (0.11–1.82)	0.267		
Lines of treatment for CRPC at the time of PET/CT				
1	1.00 (ref)	–		
≥2	1.72 (0.61–4.84)	0.30		
Prior chemotherapy				
No	1.00 (ref)	–		
Yes	1.70 (0.83–3.47)	0.14		
Site of Metastases at the time of PET/CT				
Exclusive lymph node	1.00 (ref)	–		
Bone and lymph node	2836 (0.00–15000)	0.98		
Visceral	5056 (0.00–26770)	0.21		
No of bone metastases				
<6	1.00 (ref)	–		
6–20	1.88 (0.65–5.40)	0.23		
≥20	2.44 (0.84–7.04)	0.09		
PSA at PET/CT				
≤35.5 ng/mL	1.00 (ref)	–		
>35.5 ng/mL	2.32 (1.02–5.27)	**0.043**		
ALP at PET/CT				
≤100 IU/L	1.00 (ref)	–		
>100 IU/L	0.92 (0.43–1.94)	0.827		
LDH at PET/CT				
≤236.5 IU/L	1.00 (ref)	–		
>236.5 IU/L	1.06 (0.48–2.31)	0.878		
***FDG***–***PET parameters***				
SUV max (1-unit)				
≤5.5	1.00 (ref)	–		
>5.5	1.05 (0.50–2.23)	0.882		
MTV (1-unit)				
≤53 cm^3^	1.00 (ref)	–		
>53 cm^3^	2.59 (1.13–5.90)	**0.023**		
TLG (1-unit)				
≤1820	1.00 (ref)	–		
>1820	3.59 (1.60–8.03)	**0.002**	3.90 (1.67–9.12)	**0.002**

*PSA* prostate-specific antigen, *ALP* alkaline phosphatase, *LDH* lactate dehydrogenase, *CRPC* castration-resistant prostate cancer, *SUV* standardized uptake value, *MTV* metabolic tumor volume, *TLG* total lesion glycolysis.

**Fig. 1:**
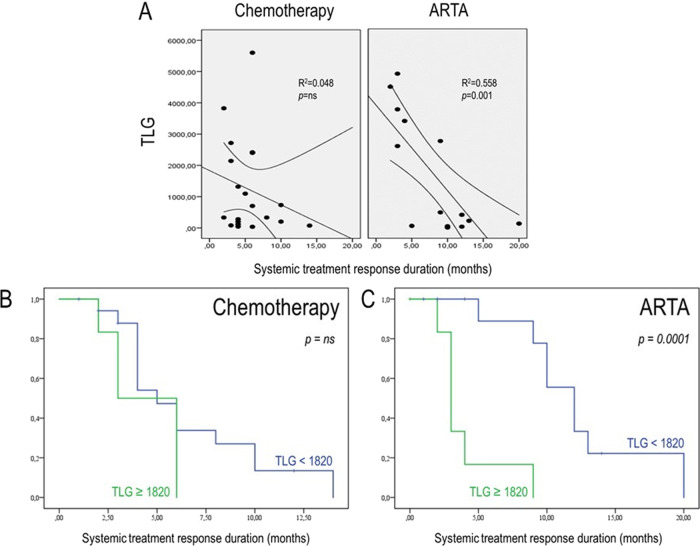
TLG in the prediction of duration of response to systemic therapy. **A** The linear correlation analyses between TLG and treatment response duration (months) in patients submitted to FDG PET/CT in the six months preceding the administration of systemic treatment with either chemotherapy or ARTA. **B**, **C** The Kaplan–Meier curves for PFS in patients with higher (green) and lower (blue) TLG levels at baseline before the administration of chemotherapy or ARTA, respectively.

**Fig. 2:**
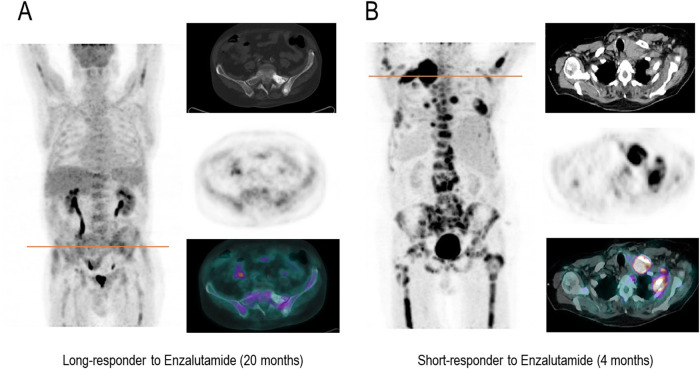
Emblematic cases of divergent response duration to the same systemic treatment in the presence of opposite TLG. In these two patients, FDG PET/CT showed a remarkable mismatch in the extent of the metabolically active metastatic burden (TLG = 59, and TLG = 5602.4 in Panels A and B, respectively), which corresponded to divergent response duration to the same systemic treatment (20 and 4 months after the first dose of Enzalutamide, respectively). On the left side of each panel Maximum intensity projection (MIP) PET images are represented, while on the right side, the axial section of CT, PET and PET/CT images of the hottest metastatic lesions (also indicated by the orange line on the MIP images) are represented from top to bottom, respectively.

Obtained cut-off values from the ROC curve for PFS for these variables are reported in Supplementary Table 4. When PSA was binarized, ARTA and Chemotherapy performed equally in the two obtained subgroups (Supplementary Fig. 5A). However, patients belonging to opposite TLG categories showed different susceptibilities to systemic treatments. Indeed, patients with lower TLG levels showed increased PFS when treated with ARTA than Chemotherapy, while these treatments performed equally in the presence of high TLG values (Supplementary Fig. 5B). This finding was also confirmed by the significant correlation between TLG and the duration of response to therapy, which was observed only in patients treated with ARTA (Fig. 1A). In other words, while PSA at the time of PET/CT could not predict the best treatment choice, the occurrence of lower TLG was able to predict higher success rates for ARTA but had no impact on chemotherapy efficacy (Fig. 1B, C). Two emblematic cases in which opposed TLG predicted divergent response duration to the same systemic treatment are reported in Fig. 2.

## Discussion

The present study supports the role of FDG-PET/CT as a prognostic biomarker in hormone-refractory PC and demonstrates its capability to predict therapy response duration before the systemic treatment administration.

Several clinical features have been used to stratify OS in mCRPC, distinguishing between high and low-risk patients [24]. Meirelles [25] and Jadvar [13, 26] firstly reported that FDG-PET/CT could predict OS in these patients, even after adjusting for other prognostic clinical confounders. The present study confirmed these findings in a larger cohort of patients as the FDG-avid metastatic burden independently predicted OS even after adjusting for PSA at the time of PET/CT.

In the clinical setting, the FDG predictive power has been employed in several ways in mCRPC. In the post-treatment phase, it has been proposed to assess therapy response using PET Response Criteria in Solid Tumors (PERCIST) in patients treated with systemic chemotherapy [27] or Radium-223 [15]. The present data extend this tool’s prognostic value to the pre-treatment setting, showing that FDG-PET/CT may also predict therapy response duration when used before the systemic treatment administration. Indeed, in the presence of a lower extent of FDG-avid disease, ARTA seems to outperform chemotherapy while performing similarly in case of higher levels of metabolically active disease. Of note, even if predicting OS, PSA value at the time of PET/CT could not improve treatment selection.

The likeliest explanation for these findings is that lower FDG uptake reflects the prevalence of well-differentiated AR‐dependent disease (potentially susceptible to ARTA). In contrast, the FDG-avid disease burden mirrors the metabolic glucose reprogramming characterizing the dedifferentiated (eventually low PSA-releasing) mCRPC and the consequent low-response rate to AR-targeted therapies. This interpretation agrees with the previous observation that glucose transporter 1 (GLUT-1) is overexpressed in AR-independent cells [28], representing a signature of PC aggressiveness [29]. Further, Fox et al. previously analyzed 133 mCRPC patients through a dual tracer PET/CT approach (FDG and 18F-Fluorodihydrotestosterone), identifying the existence of at least four different phenotypes of metastatic lesions based on a dichotomous classification of imaging findings [30]. Using histopathology as a reference, the authors observed that when FDG-avid, mCRPC lesions are less differentiated and low expressing AR [30]. Moreover, the higher was the preponderance of the FDG + /AR- disease, the poorer was the clinical outcome [30]. Similar findings have been more recently reproduced using a dual tracer approach with FDG and Prostate-Specific Membrane Antigen (PSMA) in patients undergoing 177Lu-PSMA radioligand therapy [12, 31–34].

The present study provides new elements to support these previous findings. From the methodological point of view, it proposes quantifying the disease burden rather than merely defining the presence or absence of FDG-avidity. The prognostic value of high (versus low) burden disease is already established in PC. Previous studies with bone scan and PSMA PET/CT showed that the volumetric measurement of disease provides invaluable prognostic information that could result in a better treatment individualization [35, 36]. Furthermore, both the CHAARTED and STAMPEDE clinical trials [37, 38] introduced its use as a parameter to consider in the PC’s therapeutic management. However, an agreed consensus definition of the high-burden disease currently lacks. Jadvar et al. estimated it by the sum of SUVmax of all lesions [13, 18]. SUV, the most diffuse semiquantitative parameter, may be considered a surrogate of metabolic activity, as a high value of SUV usually reflects the tumor’s aggressiveness and poor prognosis. However, it intrinsically presents some limitations, including the strict dependency on blood glucose level, the eventual radiotracer’s extravasation, the body weight, the potential residual activity in the syringe, the uptake time, the radiotracer decay, the partial volume effect, the scanner features, the protocols of acquisition and elaboration [39, 40]. For these reasons, in addition to the usefulness of SUVmax, several studies indicated that MTV and TLG could provide superior prognostic information in various cancer types [41, 42]. We quantified the disease burden using these two parameters on these bases and according to a few previous studies [15, 16, 34, 43]. This approach allowed us to confirm that MTV is a robust predictor of long-term survival in mCRPC. This is in line with previous studies showing that the higher the tumor burden volume the shorter is long-term survival (regardless of treatment choices) [35, 36]. On the other hand, TLG overcame MTV in predicting systemic treatment response duration. TLG can be considered as a compromise between morphological and metabolic features, including the tumor size and the metabolic activity at the same time. The independent predictive value of TLG suggests that not only the extent (displayed by MTV, which is one of the TLG determinants) but also the intensity of the metastatic burden’s FDG uptake concur to define the systemic therapy’s response. As FDG uptake intensity reflects the degree of metabolic glucose reprogramming, TLG might provide pathophysiological insights into the PC biology in vivo, potentially better guiding the treatment choice (once integrated with tumor volume) compared to the volumetric estimation alone.

On the clinical ground, this finding has practical implications for PC’s drug development in the era of Precision Oncology [44, 45]. Indeed, cancer heterogeneity represents a significant challenge for this approach. Previous studies highlighted that circulating tumor cells represent a promising biomarker for predicting inadequate response to ARTA, potentially improving treatment individualization [46]. However, this approach is costly and not less available. Conversely, FDG-PET/CT may represent a widely available biomarker. Whether confirmed by further studies, FDG imaging may identify subgroups of patients most likely to benefit from a specific therapy, thus optimizing treatment sequencing. On the other hand, it may open new opportunities for targeting PC glucose metabolism in addition to or instead of AR-target therapies [11].

The present study has some limitations. First, due to its multicentric nature, we must assume that the PET scanners were not cross calibrated to ensure that the same SUV was measured when the same lesion was assessed on two different PET scanners. More importantly, the retrospective design did not allow us to prevent any potential patient selection bias. Indeed, the decision to order the FDG-PET/CT scan was made by the treating oncologist and was based on medical and non‐medical considerations, including the unavailability of other more specific PET radiotracers for prostate cancer (such as choline or PSMA). On these bases, we cannot exclude that, in some cases, mCRPC patients referred to FDG-PET/CT might have been considered by the treating physician at higher risk. This might have reduced the generalizability of our findings to most prostate cancer patients. The same consideration applies to the retrospective selection of mCRPC patients submitted to FDG-PET/CT in the six months preceding systemic treatment administration, which identified a subgroup of patients with remarkably high PSA levels compared to the initial cohort. Despite these concerns, to the best of our knowledge, the present study represents the first to determine the interplay between FDG avidity and systemic treatment response duration in mCRPC. However, the current results should be considered proof-of-concept findings to be used as the baseline to design future larger and prospective studies. Indeed, a 2-arm randomized design using cross-calibrated PET scanners is needed to more robustly demonstrate whether individual patient groups defined through FDG imaging may predict distinct treatment response patterns. As a further limitation, due to the low sample size, the survival analysis was not stratified according to each ARTA or Chemotherapy agent type, considering their common mechanisms of AR signaling inhibition or disruption of microtubule function, respectively. However, results may have differed regarding their distinct mechanisms of action. More importantly, given that Cabazitaxel is administered only in previously Docetaxel-treated patients, the same category included chemotherapy-naïve and already Docetaxel-treated patients. Despite the presence of a similar percentage of chemotherapy-naïve mCRPC in the ARTA subgroup might have reduced the impact of this methodological bias, this point still needs to be addressed in the next future. Similarly, we recognize that there has been a change in the treatment landscape for mCRPC in the last years, most notably with the introduction of novel antiandrogens. Larger patient numbers, longer follow-up, and more detailed analyses of treatment-related subgroups (including patients who have received docetaxel in the castrate-sensitive phase of the disease) will be required to corroborate our preliminary observation.

## Conclusions

FDG-PET/CT provides relevant prognostic insights in hormone-refractory prostate cancer. PET-based parameters such as TLG, incorporating the estimation of both tumor metabolic activity and the extent of disease burden, can also predict systemic treatment response duration. Whether prospectively confirmed, this molecular imaging-based approach may improve treatment individualization, opening a new window on the clinical and biological mCRPC heterogeneity’s characterization.

## Supplementary information

Supplementary Materials

Supplementary Figure 1

Supplementary Figure 2

Supplementary Figure 3

Supplementary Figure 4

Supplementary Figure 5

## Data Availability

The data that support the findings of this study are available from the corresponding author upon reasonable request.

## References

[1] Morris MJ, Heller G, Bryce AH, Armstrong AJ, Beltran H, Hahn OM (2019). Alliance A031201: A phase III trial of enzalutamide (ENZ) versus enzalutamide, abiraterone, and prednisone (ENZ/AAP) for metastatic castration resistant prostate cancer (mCRPC). J Clin Oncol.

[2] McKay RR, Xie W, Fennessy FM, Zhang Z, Lis R, Rathkopf DE (2020). Results of a phase II trial of intense androgen deprivation therapy prior to radical prostatectomy (RP) in men with high-risk localized prostate cancer (PC). J Clin Oncol.

[3] Andrews JR, Ahmed ME, Karnes RJ, Kwon E, Bryce AH (2020). Systemic treatment for metastatic castrate resistant prostate cancer: does sequence matter?. Prostate.

[4] Miyake H, Sugiyama T, Aki R, Matsushita Y, Tamura K, Motoyama D (2018). Comparison of alternative androgen receptor‐axis‐targeted agent (ARATA) and docetaxel as second‐line therapy for patients with metastatic castration‐resistant prostate cancer with progression after initial ARATA in real‐world clinical practice in Japan. Clin Genitourin Cancer.

[5] Oh WK, Cheng WY, Miao R, Vekeman F, Gauthier-Loiselle M, Duh MS (2018). Real‐world outcomes in patients with metastatic castration‐resistant prostate cancer receiving second‐line chemotherapy versus an alternative androgen receptor‐targeted agent (ARTA) following early progression on a first‐line ARTA in a US community oncology setting. Urol Oncol.

[6] Matsubara N, Yamada Y, Tabata KI, Satoh T, Kamiya N, Suzuki H (2017). Comparison of sequential treatment with androgen receptor‐targeted agent followed by another androgen receptor‐targeted agent versus androgen receptor‐targeted agent followed by docetaxel in chemotherapy‐naive patients with metastatic castration‐resistant. Clin Genitourin Cancer.

[7] Oh WK, Miao R, Vekeman F, Sung J, Cheng WY, Gauthier-Loiselle M (2018). Real-world characteristics and outcomes of patients with metastatic castration-resistant prostate cancer receiving chemotherapy versus androgen receptor-targeted therapy after failure of first-line androgen receptor-targeted therapy in the community setting. Clin Genitourin Cancer.

[8] Scher HI, Morris MJ, Stadler WM, Higano C, Basch E, Fizazi K (2016). Trial design and objectives for castration-resistant prostate cancer: updated recommendations from the prostate cancer clinical trials working group 3. J Clin Oncol.

[9] Maines F, Caffo O, Veccia A, Trentin C, Tortora G, Galligioni E (2015). Sequencing new agents after docetaxel in patients with metastatic castration-resistant prostate cancer. Crit Rev Oncol Hematol.

[10] Geng H, Xue C, Mendonca J, Sun XX, Liu Q, Reardon PN (2018). Interplay between hypoxia and androgen controls a metabolic switch conferring resistance to androgen/AR-targeted therapy. Nat Commun.

[11] Wang J, Xu W, Wang B, Lin G, Wei Y, Abudurexiti M (2020). GLUT1 is an AR target contributing to tumor growth and glycolysis in castration-resistant and enzalutamide-resistant prostate cancers. Cancer Lett.

[12] Suman S, Parghane RV, Joshi A, Prabhash K, Bakshi G, Talole S (2019). Therapeutic efficacy, prognostic variables and clinical outcome of 177Lu-PSMA-617 PRLT in progressive mCRPC following multiple lines of treatment: prognostic implications of high FDG uptake on dual tracer PET-CT vis-à-vis Gleason score in such cohort. Br J Radio.

[13] Jadvar H, Desai B, Ji L, Conti PS, Dorff TB, Groshen SG (2013). Baseline 18F-FDG PET/CT parameters as imaging biomarkers of overall survival in castrate-resistant metastatic prostate cancer. J Nucl Med.

[14] Jadvar H (2016). Is there use for FDG-PET in prostate cancer?. Semin Nucl Med.

[15] Bauckneht M, Capitanio S, Donegani MI, Zanardi E, Miceli A, Murialdo R (2019). Role of baseline and post-therapy 18F-FDG PET in the prognostic stratification of metastatic castration-resistant prostate cancer (mCRPC) patients treated with radium-223. Cancers (Basel).

[16] Bauckneht M, Rebuzzi SE, Signori A, Donegani MI, Murianni V, Miceli A (2020). The prognostic role of baseline metabolic tumor burden and systemic inflammation biomarkers in metastatic castration-resistant prostate cancer patients treated with radium-223: a proof of concept study. Cancers (Basel).

[17] Jadvar H, Velez EM, Desai B, Ji L, Colletti PM, Quinn DI (2019). Prediction of time to hormonal treatment failure in metastatic castration-sensitive prostate cancer with 18F-FDG PET/CT. J Nucl Med.

[18] Jadvar H (2013). Imaging evaluation of prostate cancer with 18F-fluorodeoxyglucose PET/CT: utility and limitations. Eur J Nucl Med Mol Imaging.

[19] AIOM Guidelines on Prostate Cancer 2019. https://www.aiom.it/wp-content/uploads/2019/10/2019_LG_AIOM_Prostata.pdf. Last accessed on 20/03/2021

[20] Boellaard R, Delgado-Bolton R, Oyen WJ, Giammarile F, Tatsch K, Eschner W (2015). FDG PET/CT: EANM procedure guidelines for tumour imaging: version 2.0. Eur J Nucl Med Mol Imaging.

[21] Kruse V, Mees G, Maes A, D’Asseler Y, Borms M, Cocquyt V (2015). Reproducibility of FDG PET based metabolic tumor volume measurements and of their FDG distribution within. Q J Nucl Med Mol Imaging.

[22] Epstein JI, Egevad L, Amin MB, Delahunt B, Srigley JR, Humphrey PA (2016). The 2014 International Society of Urological Pathology (ISUP) consensus conference on gleason grading of prostatic carcinoma: definition of grading patterns and proposal for a new grading system. Am J Surg Pathol.

[23] Parker C, Nilsson S, Heinrich D, Helle SI, O’Sullivan JM, Fosså SD (2013). Alpha emitter radium-223 and survival in metastatic prostate cancer. N. Engl J Med.

[24] Halabi S, Lin CY, Kelly WK, Fizazi KS, Moul JW, Kaplan EB (2014). Updated prognostic model for predicting overall survival in first-line chemotherapy for patients with metastatic castration-resistant prostate cancer. J Clin Oncol.

[25] Meirelles GS, Schöder H, Ravizzini GC, Gönen M, Fox JJ, Humm J (2010). Prognostic value of baseline [18F] fluorodeoxyglucose positron emission tomography and 99mTc-MDP bone scan in progressing metastatic prostate cancer. Clin Cancer Res.

[26] Jadvar H, Groshen SG, Quinn DI (2015). Association of overall survival with glycolytic activity of castrate-resistant prostate cancer metastases. Radiology.

[27] Velez EM, Desai B, Ji L, Quinn DI, Colletti PM, Jadvar H (2020). Comparative prognostic implication of treatment response assessments in mCRPC: PERCIST 1.0, RECIST 1.1, and PSA response criteria. Theranostics.

[28] Vaz CV, Alves MG, Marques R, Moreira PI, Oliveira PF, Maia CJ (2012). Androgen-responsive and nonresponsive prostate cancer cells present a distinct glycolytic metabolism profile. Int J Biochem Cell Biol.

[29] Qu W, Ding SM, Cao G, Wang SJ, Zheng XH, Li GH (2016). miR-132 mediates a metabolic shift in prostate cancer cells by targeting Glut1. FEBS Open Bio.

[30] Fox JJ, Gavane SC, Blanc-Autran E, Nehmeh S, Gönen M, Beattie B (2018). Positron emission tomography/computed tomography-based assessments of androgen receptor expression and glycolytic activity as a prognostic biomarker for metastatic castration-resistant prostate cancer. JAMA Oncol.

[31] Michalski K, Ruf J, Goetz C, Seitz AK, Buck AK, Lapa C, et al. Prognostic implications of dual tracer PET/CT: PSMA ligand and [18F]FDG PET/CT in patients undergoing [177Lu]PSMA radioligand therapy. Eur J Nucl Med Mol Imaging. (2020). 10.1007/s00259-020-05160-8. 10.1007/s00259-020-05160-8 PMC8113196 33336265

[32] Thang SP, Violet J, Sandhu S, Iravani A, Akhurst T, Kong G (2019). Poor outcomes for patients with metastatic castration-resistant prostate cancer with low prostate-specific membrane antigen (PSMA) expression deemed ineligible for (177)Lu-labelled PSMA radioligand therapy. Eur Urol Oncol.

[33] Wang B, Liu C, Wei Y, Meng J, Zhang Y, Gan H (2020). A prospective trial of 68Ga-PSMA and 18F-FDG PET/CT in nonmetastatic prostate cancer patients with an early PSA progression during castration. Clin Cancer Res.

[34] Ferdinandus J, Violet J, Sandhu S, Hicks RJ, Ravi Kumar AS, Iravani A (2020). Prognostic biomarkers in men with metastatic castration-resistant prostate cancer receiving [177Lu]-PSMA-617. Eur J Nucl Med Mol Imaging.

[35] Mota JM, Armstrong AJ, Larson SM, Fox JJ, Morris MJ (2019). Measuring the unmeasurable: automated bone scan index as a quantitative endpoint in prostate cancer clinical trials. Prostate Cancer Prostatic Dis.

[36] Gafita A, Bieth M, Krönke M, Tetteh G, Navarro F, Wang H (2019). qPSMA: semiautomatic software for whole-body tumor burden assessment in prostate cancer using 68Ga-PSMA11 PET/CT. J Nucl Med.

[37] Kyriakopoulos CE, Chen YH, Carducci MA, Liu G, Jarrard DF, Hahn NM (2018). Chemohormonal Therapy in metastatic hormone-sensitive prostate cancer: long-term survival analysis of the randomized phase III E3805 CHAARTED trial. J Clin Oncol.

[38] Parker CC, James ND, Brawley CD, Clarke NW, Hoyle AP, Ali A (2018). Radiotherapy to the primary tumour for newly diagnosed, metastatic prostate cancer (STAMPEDE): a randomised controlled phase 3 trial. Lancet.

[39] Keyes JW (1995). SUV: standard uptake or silly useless value?. J Nucl Med.

[40] Kostakoglu L, Chauvie S (2018). Metabolic Tumor Volume Metrics in Lymphoma. Semin Nucl Med.

[41] Zhang H, Wroblewski K, Appelbaum D, Pu Y (2013). Independent prognostic value of whole-body metabolic tumor burden from FDG-PET in non-small cell lung cancer. Int J Comput Assist Radio Surg.

[42] Zhang C, Liao C, Penney BC, Appelbaum DE, Simon CA, Pu Y (2015). Relationship between overall survival of patients with non-small cell lung cancer and whole-body metabolic tumor burden seen on postsurgical fluorodeoxyglucose PET images. Radiology.

[43] Wibmer AG, Morris MJ, Gonen M, Zheng J, Hricak H, Larson SM, et al. Quantification of metastatic prostate cancer whole-body tumor burden with FDG PET parameters and associations with overall survival after first-line abiraterone or enzalutamide: a single-center retrospective cohort study. J Nucl Med. (2021). 10.2967/jnumed.120.256602. 10.2967/jnumed.120.256602 PMC8833874 33419944

[44] Moscow JA, Fojo T, Schilsky RL (2018). The evidence framework for precision cancer medicine. Nat Rev Clin Oncol.

[45] Jameson JL, Longo DL (2015). Precision medicine−personalized, problematic, and promising. N Engl J Med.

[46] Antonarakis ES, Lu C, Wang H, Luber B, Nakazawa M, Roeser JC (2014). AR-V7 and resistance to enzalutamide and abiraterone in prostate cancer. N. Engl J Med.

