# Supplementary material for: The prognostic power of 18F-FDG PET/CT extends to estimating systemic treatment response duration in metastatic castration-resistant prostate cancer (mCRPC) patients
Source: Prostate Cancer Prostatic Dis. 2021 May 19;24(4):1198–207. doi: 10.1038/s41391-021-00391-8 (PMC8616756; doi:10.1038/s41391-021-00391-8)
Supplement: Supplementary file 1 — Supplementary Materials [file 41391_2021_391_MOESM1_ESM.docx]

**Supplementary Table 1. Overview of participating centers and equipment**

| **PET/CT center** | **Brescia** | **Brescia (2)** | **Genova** | **Reggio Emilia** | **Reggio Emilia (2)** | **Messina** |
| --- | --- | --- | --- | --- | --- | --- |
| **Model and Manufacturer** | GE Healthcare  Discovery STE | GE Healthcare  Discovery 690 | Siemens  Hirez  Biograph 16 | GE Healthcare  Discovery STE | GE Healthcare  Discovery MI | Philips  Gemini  TF 16 |
| **Spatial resolution** | | | | | | |
| In-plane FWHM (mm) | 5.55 | 4.74 | 5.8 | 5.55 | 4.6 | 5.8 |
| Slice thickness | 3.27 | 2.79 | 3.75 | 3.27 | 2.79 | 4.0 |
| **Axial Field of View (mm)** | 155 | 157 | 162 | 157 | 200 | 180 |

FWHM, Full width at half-maximum height

**Supplementary Table 2**: **Cut-off values calculated using ROC curve analysis considering the entire population (n=114).**

| ROC curve analysis for OS | | | | | | |
| --- | --- | --- | --- | --- | --- | --- |
| **Parameter** | AUC (95% CI) | *p value* | Cut-off value | Youden Index | Sensitivity | Specificity |
| PSA at diagnosis (ng/mL) | 0.530 (0.397-0.663) | 0.643 | 22.50 | 0.03 | 0.61 | 0.42 |
| ALP at diagnosis (IU/L) | 0.624 (0.295-0.953) | 0.340 | 67.50 | 0.51 | 0.94 | 0.57 |
| LDH at diagnosis (IU/L) | 0.679 (0.418-0.939) | 0.172 | 223.50 | 0.01 | 0.64 | 0.37 |
| PSA at PET/CT (ng/mL) | 0.722 (0.508-0.936) | 0.119 | 58.30 | 0.50 | 0.50 | 1.00 |
| ALP at PET/CT (IU/L) | 0.556 (0.185-0.926) | 0.697 | 45.50 | 0.50 | 1.00 | 0.50 |
| LDH at PET/CT (IU/L) | 0.602 (0.482-0.723) | 0.097 | 214.50 | 0.19 | 0.67 | 0.52 |
| SUVmax | 0.431 (0.320-0.542) | 0.215 | 6.90 | 0.01 | 0.47 | 0.54 |
| MTV (cm^3^) | 0.630 (0.526-0.734) | 0.019 | 325.97 | 0.19 | 0.35 | 0.83 |
| TLG | 0.628 (0.523-0.732) | 0.021 | 844.86 | 0.33 | 0.50 | 0.83 |

**Supplementary Table 3: Pre- systemic treatment cohorts characteristics (n=44)**

|  | **General** (n=44) | **ARTA** (n=20) | **Chemotherapy** (n=24) | *p* |
| --- | --- | --- | --- | --- |
| **Age** | 72.7±7.7 | 72.3±7.7 | 73.1±7.9 | ns |
|  |  |  |  |  |
| **Gleason Score at diagnosis** |  |  |  |  |
| *≤ 7* | 21 (47.7%) | 10 (50%) | 11 (45.8%) | ns |
| ≥ *8* | 23 (52.3%) | 10 (50%) | 13 (54.2%) | ns |
| *Missing data* | - | - | - | - |
|  |  |  |  |  |
| **Metastatic disease at diagnosis** |  |  |  |  |
| *Yes* | 41 (93.2%) | 19 (95%) | 22 (92%) | ns |
| *No* | 3 (6.8%) | 1 (5%) | 2 (8%) | ns |
| *Missing data* | - | - | - | - |
|  |  |  |  |  |
| **Lab tests at diagnosis** |  |  |  |  |
| *PSA (ng/mL)* | 532.1±2123.7 | 976.8±3216.1 | 193.1±287.6 | ns |
| *AP (IU/L)* | 171.1±112.2 | 164.6±105.4 | 175.7±124.9 | ns |
| *LDH (IU/L)* | 258.5±138.4 | 224.2±135.7 | 283.1±145.5 | ns |
|  |  |  |  |  |
| ***Lines of treatment for CRPC*** |  |  |  |  |
| *1* | 13 (29.5%) | 7 (15.9%) | 6 (13.6%) | ns |
| *2* | 5 (11.4%) | 1 (2.3%) | 4 (9.1%) | ns |
| *≥2* | 25 (56.8 %) | 12 (27.3%) | 13 (29.5%) | ns |
| *Missing data* | 1 (2.3%) | 0 (0%) | 1 (2.3%) | - |
|  |  |  |  |  |
| **Prior Chemotherapy** |  |  |  |  |
| *Yes* | 26 (59.1%) | 10 (22,7%) | 16 (36.4%) | ns |
| *No* | 18 (40.9%) | 10 (22.7%) | 8 (18.2%) | ns |
|  |  |  |  |  |
| **Lab tests at the time of PET/CT** |  |  |  |  |
| *PSA (ng/mL)* | 458.1±788.1 | 592.1±984.3 | 347.5±580.2 | ns |
| *ALP (IU/L)* | 164.1±176.4 | 156.1±144.3 | 171.7±205.9 | ns |
| *LDH (IU/L)* | 264.1±143.1 | 286.8±172.4 | 243.8±112.2 | ns |
|  |  |  |  |  |
| **Site of Metastases at the time of PET/CT** |  |  |  |  |
| *Exclusive lymph node metastases* | 1 (2.3%) | 1 (2.3%) | 0 (0%) | ns |
| *Bone and lymph node metastases* | 32 (72.7%) | 15 (34.1%) | 17 (38.6%) | ns |
| *Visceral metastases* | 11 (25%) | 4 (9.1%) | 7 (15.9%) | ns |
|  |  |  |  |  |
| **N° of bone metastases** |  |  |  |  |
| *<6* | 8 (18.2%) | 4 (9.1%) | 4 (9.1%) | ns |
| *6-20* | 12 (27.3%) | 4 (9.1%) | 8 (18.2%) | ns |
| *>20* | 10 (22.7%) | 5 (11.4%) | 5 (11.4%) | ns |
| *Missing data* | 14 (31.8%) | 7 (15.9%) | 7 (15.9%) | ns |
|  |  |  |  |  |
| **FDG PET/CT parameters** |  |  |  |  |
| *SUVmax of the hottest lesion* | 7.6±3.5 | 6.8±2.9 | 8.3±3.9 | ns |
| *MTV (cm3)* | 311.6±397.5 | 343.7±474.2 | 284.9±328.7 | ns |
| *TLG* | 1285.9±1563.5 | 1405.6±1777.4 | 1186.2±1392 | ns |

PSA: prostate specific antigen; ALP: alkaline phosphatase LDH: lactate dehydrogenase; CRPC: castration-resistant prostate cancer; SUV: standardized uptake value; MTV: metabolic tumor volume; TLG: total lesion glycolysis; ARTA: Androgen Receptor-Targeted Agents; ns: not significant

**Supplementary Table 4**: **Cut-off values calculated using ROC curve analysis considering patients submitted to FDG PET/CT imaging in the six months preceding systemic treatment initiation (n=44).**

| ROC curve analysis for PFS | | | | | | |
| --- | --- | --- | --- | --- | --- | --- |
| **Parameter** | AUC (95% CI) | *p value* | Cut-off value | Youden Index | Sensitivity | Specificity |
| PSA at diagnosis (ng/mL) | 0.361 (0.138-0.584) | 0.215 | 18.10 | 0.15 | 0.60 | 0.55 |
| ALP at diagnosis (IU/L) | 0.100 (0.000-0.306) | 0.086 | 150.00 | 0.40 | 0.40 | 1.00 |
| LDH at diagnosis (IU/L) | 0.100 (0.000-0.286) | 0.086 | 262.00 | 0.40 | 0.40 | 1.00 |
| PSA at PET/CT (ng/mL) | 0.456 (0.241-0.672) | 0.679 | 35.50 | 0.11 | 0.71 | 0.40 |
| ALP at PET/CT (IU/L) | 0.332 (0.118-0.545) | 0.150 | 100.00 | 0.03 | 0.41 | 0.62 |
| LDH at PET/CT (IU/L) | 0.457 (0.191-0.722) | 0.715 | 236.50 | 0.08 | 0.46 | 0.62 |
| SUVmax | 0.491 (0.264-0.718) | 0.933 | 5.50 | 0.20 | 0.70 | 0.50 |
| MTV (cm^3^) | 0.518 (0.318-0.718) | 0.867 | 53.00 | 0.27 | 0.67 | 0.60 |
| TLG | 0.548 (0.355-0.742) | 0.647 | 1820.00 | 0.24 | 0.34 | 0.90 |

**Supplementary Figure Titles and Legends.**

**Supplementary Figure 1: Kaplan Meier survival function of the study cohort.**

**Supplementary Figure 2: Prognostic role of clinical, biochemical and FDG PET/CT-derived parameters.** Kaplan Meier curves for the number of bone metastases (Panel A), PSA (Panel B), ALP (Panel C), MTV (Panel D), and TLG (Panel E) in the prediction of OS considering the entire population of the study. The cut-off values for continuous variables were defined by means of the Youden index from the ROC curve for OS data, as shown in Supplementary Table 3.

**Supplementary Figure 3: Selection process of the cohort of 44 patients from the initial 114 mCRPC.**

**Supplementary Figure 4: OS and PFS Kaplan Meier curves according to the administered systemic treatment.** Panels A and B respectively display the Kaplan Meier curves in the prediction of OS and PFS in patients submitted to FDG PET/CT before administering systemic treatment (ARTA, blue or Chemotherapy, green).

**Supplementary Figure 5: Biochemical and FDG PET/CT-derived parameters in the prediction of systemic treatment response duration.** Kaplan Meier curves for PSA (Panel A), and TLG (Panel B) in the prediction of systemic treatment duration (PFS) considering patients submitted to FDG PET/CT imaging in the six months preceding the administration of Chemotherapy (green curves) or ARTA (blue curves). The cut-off values were defined by means of the Youden index from the ROC curve for PFS data, as shown in Supplementary Table 4.
