# Supplementary figures and images for: The prognostic power of 18F-FDG PET/CT extends to estimating systemic treatment response duration in metastatic castration-resistant prostate cancer (mCRPC) patients
Source: Prostate Cancer Prostatic Dis. 2021 May 19;24(4):1198–207. doi: 10.1038/s41391-021-00391-8 (PMC8616756; doi:10.1038/s41391-021-00391-8)

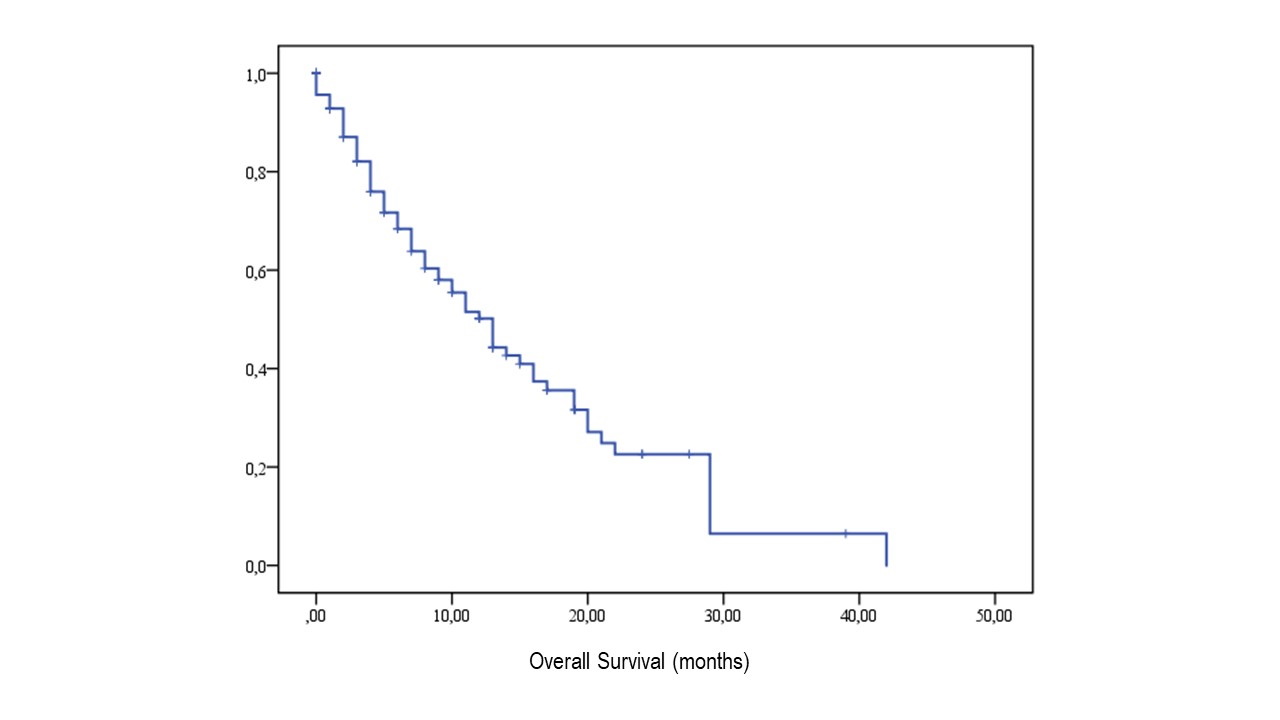

Supplement: Supplementary file 2 — Supplementary Figure 1 [file 41391_2021_391_MOESM2_ESM.jpg]

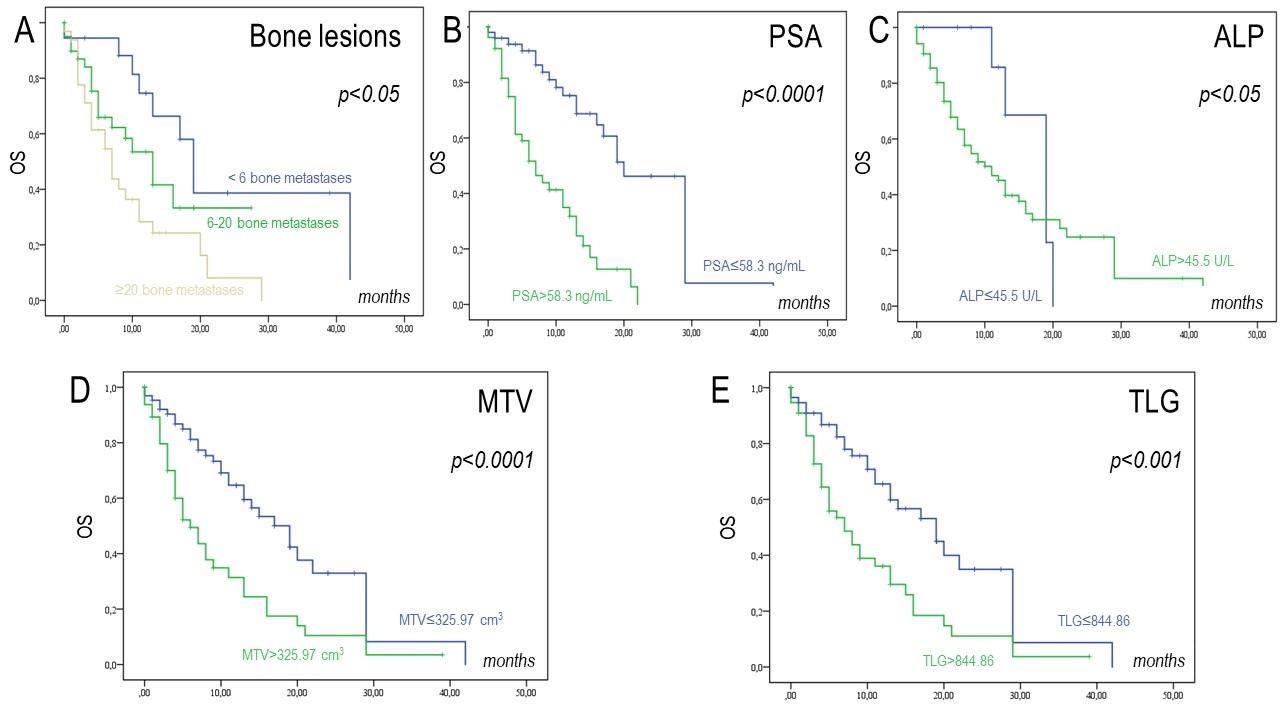

Supplement: Supplementary file 3 — Supplementary Figure 2 [file 41391_2021_391_MOESM3_ESM.jpg]

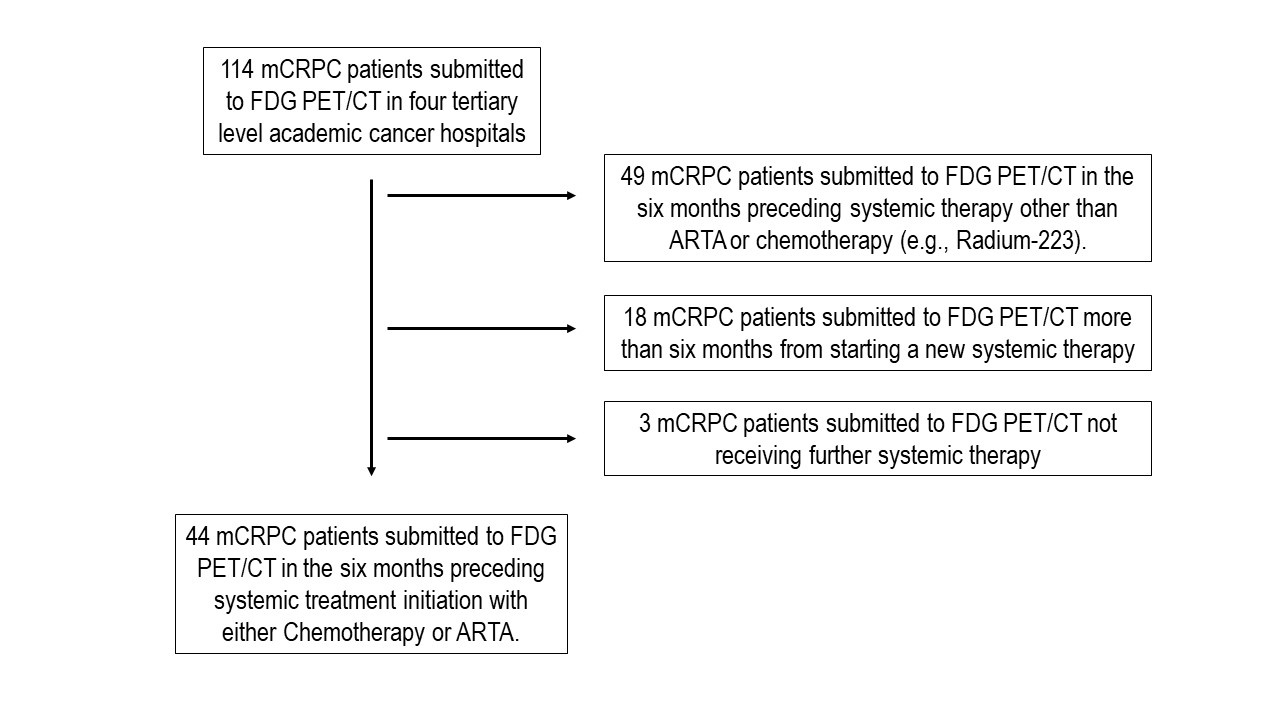

Supplement: Supplementary file 4 — Supplementary Figure 3 [file 41391_2021_391_MOESM4_ESM.jpg]

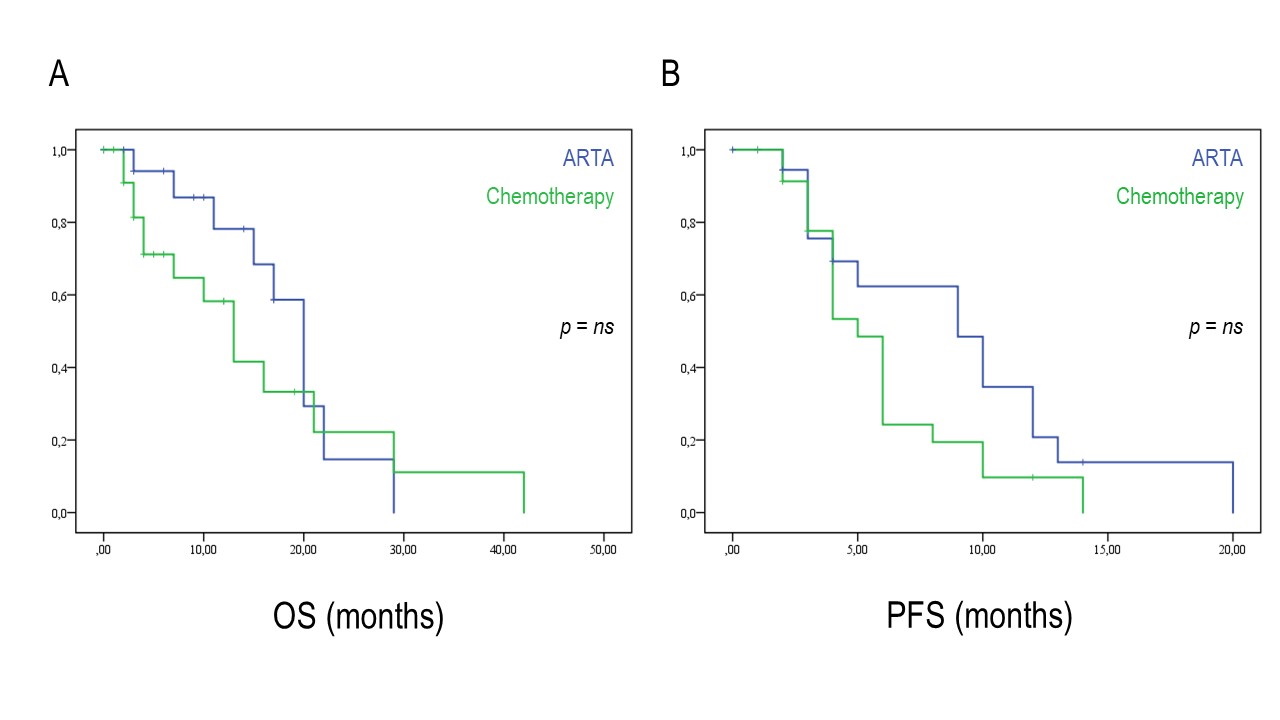

Supplement: Supplementary file 5 — Supplementary Figure 4 [file 41391_2021_391_MOESM5_ESM.jpg]

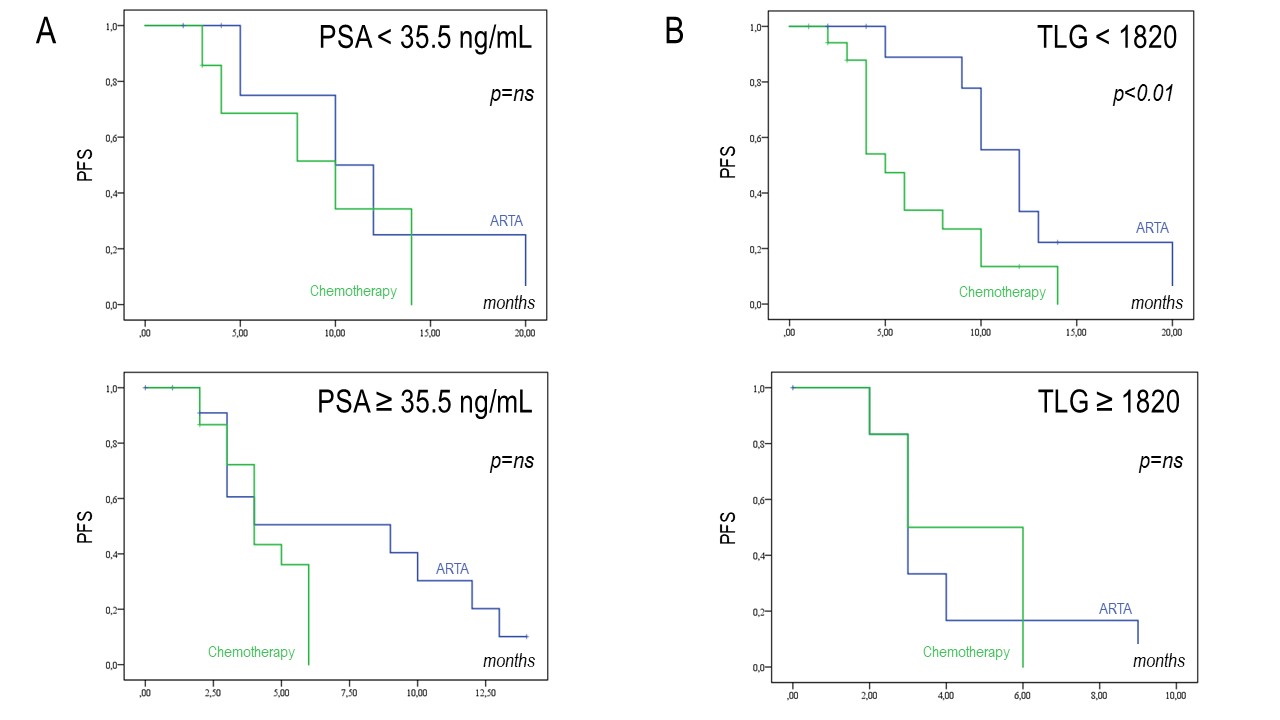

Supplement: Supplementary file 6 — Supplementary Figure 5 [file 41391_2021_391_MOESM6_ESM.jpg]
